# The Effect of Different Oak Products Used during Fermentation and Ageing on the Sensory Properties of a White Wine over Time

**DOI:** 10.3390/foods9091220

**Published:** 2020-09-02

**Authors:** Anri Botha, Wessel du Toit, Jeanne Brand, Martin Kidd, Niël Groenewald

**Affiliations:** 1 Department of Viticulture and Oenology, South African Grape and Wine Research Institute, Stellenbosch University, Private Bag X1, Matieland 7602, South Africa; anri91@gmail.com (A.B.); jeanne@sun.ac.za (J.B.); 2 Centre for Statistical Consultation, Stellenbosch University, Stellenbosch 7599, South Africa; mkidd@sun.ac.za; 3 DGB (PTY) Ltd., Lady Loch Weg, Wellington 7654, South Africa; DPGroenewald@libertasvineyards.co.za

**Keywords:** Chenin blanc wine, oak wood, wine maturation

## Abstract

The sensorial evaluation of Chenin blanc wine produced with different types of oak wood treatments over time has not been investigated before. The main aim of this study was to assess the sensory profile, using a trained panel, of a South African Chenin blanc wine fermented and matured in old barrels, three types of new oak barrels, two types of oak staves as well as a stainless steel tank over time, which has not been done before. Results indicate mainly separation between the stainless-steel tank/old barrel treatments from the stave and new barrel treatments, with separation between the latter two treatments also being found. More fruity descriptors were used in the stainless-steel tank and old barrel treatments, with oak-related descriptors being used in the stave and new barrel treatments. Separation between among the new barrel and stave treatments were also noted, which was still reflected during bottle maturation. These results indicate that winemakers need to take cognizance of the sensorial differences induced by barrel and stave maturation in white wines and how these evolve over time.

## 1. Introduction

Chenin blanc grapes have been described as having a neutral taste, which is sometimes also found in the corresponding wines [1]. However, guava and apple descriptors have also been reported in young Chenin blanc wines [2]. A number of winemaking practices that might change white wine aroma in general have been investigated. These include oxidative and reductive winemaking as well as SO_2_ and ascorbic acid must additions [3,4]. Extended skin maceration and effects of natural and different *Saccharomyces* and non *Saccharomyces* on Chenin blanc in particular have also been assessed [5,6]. General effects of oak wood fermentation and maturation have been investigated in certain white wines, but not in detail for Chenin blanc wine [7,8,9,10,11].

Organoleptic improvements of wines due to fermentation and maturation in oak barrels are often achieved [12]. Contact with oak wood enriches the wine with new compounds, which affects the sensory composition of the wine. These compounds can be divided into polyphenols that can affect the taste and structure of the wines, and volatile compounds that can influence the aroma of the wine. A number of factors can influence the concentrations at which these compounds are extracted during oak maturation. Levels of these compounds and their precursors in the wood, the rate of their release or consumption by further chemical or biochemical transformation, or time of storage and temperature might all influence this [13].

In the past, wooden barrels were mostly used for oak maturation, however, the use of alternative oak products, such as oak staves or oak chips in old barrels or stainless-steel tanks have increased [14]. This is due to barrels requiring a lot of space in the cellar, as well as being expensive with a limited lifetime [15]. Other alternative products for oak maturation include oak powder, beans, shavings or barrel inserts [16]. How volatiles changes in wine matured with alternative oak products, (sometimes in combination with micro-oxygenation) to mimic those conditions achieved by traditional barrel ageing, have been the subject of several studies [9,16,17,18,19]. However, not much of this research focused on Chenin blanc wines, especially on how barrels compare with alternative oak treatments, with Chardonnay and Sauvignon blanc wines being investigated [11]. More research on the sensory evolution of Chenin blanc wines matured in alternative oak treatments compared to oak barrels is thus also required.

The main aim of this study was to assess the influence of different oak regimens on the sensory properties of a Chenin blanc wine during three intervals using a rapid sensory method [20,21].

## 2. Materials and Methods

### 2.1. Grape Origin and Vinification Procedures

Chenin blanc grapes (2014 vintage) of the Phisantekraal Vineyards situated in the Tygerberg region in the Western Cape of South Africa were used. The grapes were harvested mechanically, with 40 mg/L metabisulphite and 20 mg/L ascorbic acid (Protea chemicals, South Africa) added and transported to the Bellingham pressing cellar (Wellington, South Africa).

At delivery to the cellar’s crushing pan, a further 30 mg/L of metabisulphite and 40 mg/L of ascorbic acid were added to the grapes. At destemming of the grapes, 0.5 g/L tartaric acid (Brenn-O-Kem, South Africa) and 20g/ton pectolytic enzyme (Lafazym Extract, Laffort, South Africa) were added to the grapes. The destemmed grapes were then cooled to 12 °C before going into the pneumatic press. No skin contact was applied and the free run juice separated from the press juice. The mash was pressed until a pH change of 0.2 occurred. A juice recovery of 616 tons/L was obtained.

Nitrogen gas sparging was used to ensure reductive conditions for the movement of the bulk free run juice. In the settling tank, with a cooling temperature set at 0 °C, the total SO_2_ and titratable acidity (TA) was adjusted to 40 mg/L and 7 g/L, respectively. The pH and Balling (°B) of the juice were 3.35 and 25.1, respectively. Settling enzyme at a dosage of 3 g/hL was also added (Lafazym CL, Laffort, South Africa). After 48 h, 8360 L of juice was racked to the fermentation tank along with approximately 1% of the fine lees.

The juice’s temperature in the fermentation tank was increased to 12 °C and inoculated with a yeast mixture of L2056, CY3079, D47 and D254 (Lallemand, Lavin, South Africa) (Lallemand, Lalvin, South Africa) at a dosage of 30 g/hL. This specific yeast mixture is often used for all barrel fermented white wines at Bellingham, due to it being able to ferment at higher temperatures without producing any off flavours. Each day the temperature and Balling was monitored. After 2 and 4 days of fermentation the must was supplemented with 15 g/hL of Fermaid-K (Lallemand, Lalvin, South Africa), which is a nitrogen supplement with added complex nutrients. The must was then transferred to the respective maturation vessels after 3 Balling of sugar was consumed by the yeast. This was done to first obtain a homogenous fermentation rate and must mixture in all the containers once the wood treatments started. Before filling, all barrels were prepared by adding cold water and a sulphur strip (Wine machinery, South Africa) was burned in the old barrels one day before filling.

All the wines received an addition of 30 mg/L SO_2_ at the end of alcoholic fermentation (residual sugar ˂4 g/L). The barrels were filled completely with wine from the original tank, and left to mature in at a constant 16 °C and a humidity that varied between 65 and 75%. The wine in the original tank was moved to a smaller tank and stored at the same temperature (16 °C) for the duration of the trial.

### 2.2. Wood Treatments

The fermentation and maturation trial consisted of four main experimental treatments (Table 1). The first and second treatments consisted of a tank control (TC) containing 2000 L wine (serving as the unoaked control) and the barrel controls (BC) consisting of the same lot of 4-year-old barrels from the same cooper (in which previously the same four wines were consecutively matured for one year each).

The new barrel treatment consisted of three types of new barrels (B1 to B3). These barrels were all produced from French wood but from different coopers, but with the same toasting level (medium toasting).

The stave treatments (French wood, medium toasting) consisted of two stave treatments (S1, dimensions: 35 × 3.2 × 4 cm and S2, dimensions: 45 × 2.5 × 1 cm). The inserts were added to old barrels of the same lot (cooperage and age) as those used in the BC treatment. The wines matured in the old oak barrels (BC) thus served as a control for the stave treatments to investigate the influence of an old barrel with and without the addition of staves. The barrel inserts replicated the effect of a new barrel and had 40% of the internal surface area of a 300-L barrel [16]. All of the treatments were performed in triplicate, except for the unoaked tank, of which only one biological repeat was available due to cellar space limitations.

### 2.3. Wine Sampling and Storage

After 4 months maturation, 10 L of wine was sampled into a 10-L canister from every treatment’s repeats. The barrels were then subsequently filled with the same wine from a stainless-steel tank. The free SO_2_ was also adjusted to 35 mg/L with Sterisol (EnolTech, EVER INTEC, Australia). The samples were transported to the experimental cellar at the Department of Viticulture and Oenology, University of Stellenbosch. The free SO_2_ levels were measured for every canister and increased to 35 mg/L with a 2.5% SO_2_ solution if required. The canisters were stored at −4 °C for two weeks before the wine was bottled in 750 mL green Burgundy bottles (Consol, XPRS, South Africa) and closed with saranex screw caps (CDS Vintec, South Africa). After bottling, the wines were stored at a constant 4 °C until the time of sensory analysis. This was done to prevent sensorial changes from happening.

After 9 months of maturation, samples were again taken using the same protocol as described for the 4-month sampling, except that 20-L samples were taken. A part of this, wine was used for immediate sensory analysis, with the rest kept at a constant 15 °C for another 6 months for further bottle maturation, which is a normal bottle ageing temperature.

### 2.4. Sensory Evaluation

Sensory evaluation was conducted on the wines at three different maturation stages: after 4 months oak maturations, after 9 months oak maturation, and after 9 months oak maturation and 6 months bottle ageing. These stages were designed to represent two typical oak aging periods used in the wine industry. Bottle ageing was incorporated because commercial cellars frequently mature wine in bottles before releasing those wines into the market. Nineteen Chenin blanc wines were evaluated by a trained panel. The Pick-*K*-attributes method, a variant of Check-All-That-Apply (CATA) was used. Panellists were asked to pick the *K* attributes that describe the product the best [22] after receiving trained with reference standards that represented each term on the list.

### 2.5. Pick-K-Attributes Using a Trained Panel

#### 2.5.1. Panel

At every maturation stage, a panel of 20 trained panellists performed the sensory analysis. Panellist recruitment was dependent on the availability of the individual panellists for the scheduled sensory testing sessions for each maturation stage. The panels used to evaluate the wines at the three maturation stages did not consist of exactly the same people, but all the panellists received the same training. The panel that evaluated the wines after 4 months consisted of 15 females and 5 males. After 9 months of barrel aging and 6 months bottle ageing, 16 females and 4 males evaluated the wines. The ages of the panellists ranged from 22 to 58 years, with an average of 26.

#### 2.5.2. Panel Training

The panel received 13 sessions of general training according to the method suggested by [22]. Each panellist attended an hour of training once a week for 13 weeks. Every training session consisted of two parts. Panellists were presented with 12–16 aroma standards, during the first part of the training session. They had to smell and identify each standard. Finally, during the second part of the session, three to four wines similar to the project were presented. Judges were asked to smell the wines and pick three to five descriptors from the list to describe the most prominent aromas of the wines. Wines were discussed while the panel leader highlighted frequently used descriptors. The list of descriptors used during the training period consisted of the descriptors on the Chenin blanc aroma wheel (refer to Botha, 201) together with descriptors selected by wine industry experts during a screening session conducted prior to the sensory training (Appendix A Table A1). One week prior to testing, the panel received Chenin blanc-specific aroma standards during training (Appendix A Table A1) as well as four wines from this project; one from each main experimental treatment in this maturation trail (TC, BC, B or S).

#### 2.5.3. Evaluation

**Figure A1:**
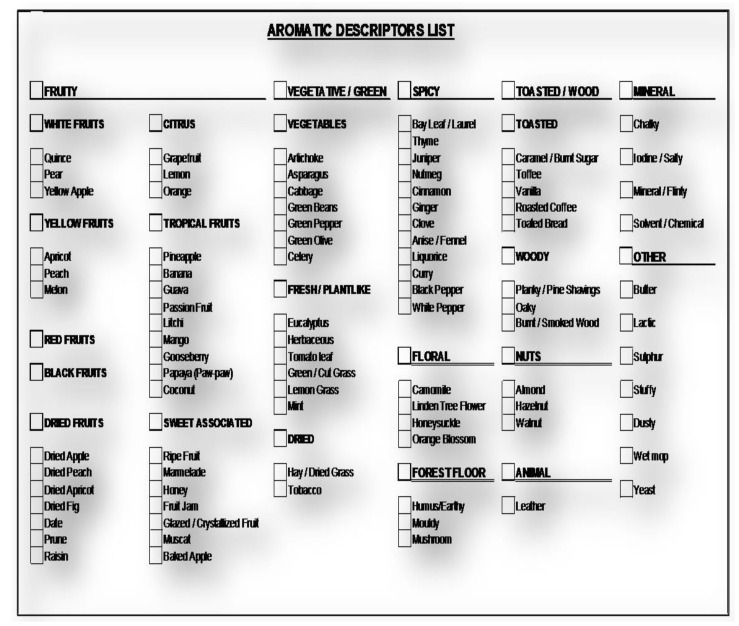
Tasting sheet used by the panel during the different stages investigated.

The trained panel evaluated a single sensory modality, namely wine aroma. Sensory evaluations were conducted in duplicate in a laboratory with individual booths, controlled temperature (20 ± 2 °C) and light conditions (ISO NORM 8589, 1988) secluded from strong odours and extraneous noise [23]. The wines were stored at 4 °C prior to evaluation, were removed from the fridge a day before testing and was stored at 20 °C until the tasting. The wines were poured (30 mL per sample) in black standard international tasting glasses (ISO NORM, 1977) and covered with Petri dish lids 1 h before testing. Samples were labelled with random three-digit codes. The serving order was randomized across the judges according to a Williams Latin-square design. To minimize sensory fatigue, three flights were presented with 6 wines in the first and 7 in the second and third flights. The panellists were asked to pick between 3 and 5 descriptors that best describe the aroma of each sample from the provided list (Figure A1). A 10-min break was enforced between flights to limit the sensory fatigue of the judges.

### 2.6. Statistical Analysis

Paper ballots were used to capture sensory data, where after it was entered into Microsoft Excel 2013 (www.microsoft.co/excel). Statistical analysis was performed in STATISTICA v. 12^®^ (www.StatSoft.com) following the protocol suggested for the analyses of frequency data as described by [21]. Contingency tables were constructed with wine samples as objects in the rows, and attributes as the variables in the columns. The frequency of citations of each attribute for each specific wine sample was determined by counting the number of judges that cited each attribute for each wine. Semantic and linguistic combination of attributes were performed by grouping similar attributes from the same aroma family where less than 20% of the panel cited one of the attributes. When a synonym could not be found the attribute was deleted. Semantic and linguistic attribute grouping was performed by two sensory scientists and two oenologists. Correspondence analysis (CA) was applied to the contingency tables.

## 3. Results and Discussion

The Balling (°B) and fermentation temperature of each fermentation vessel were recorded daily. After 20 days of alcoholic fermentation, all the wines were fermented to dryness (residual sugar levels lower than 4 g/L).

**Figure A2:**
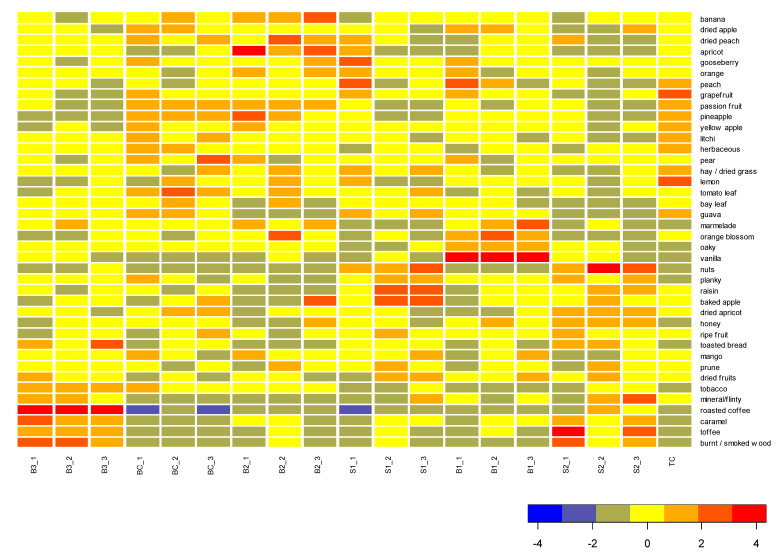
Heat maps constructed based on the standardized residuals that were generated by the correspondence analyses of individual repeats obtained with the trained panel (*n* = 20) after 4 months of oak maturation. The more negative (i.e., the more “blue” on the heatmap) the residuals, the more indication that the correspondence (co-occurrence) between the items are less than what would be expected under the assumption of complete random occurrence. And vice versa, the more positive (i.e., the more red on the heatmap) the residuals, the more indication that the co-occurrence are more that expected under complete random occurrence. TC: Tank control, BC: Barrel control, B: New barrel treatments, S: Stave treatments.

**Figure A3:**
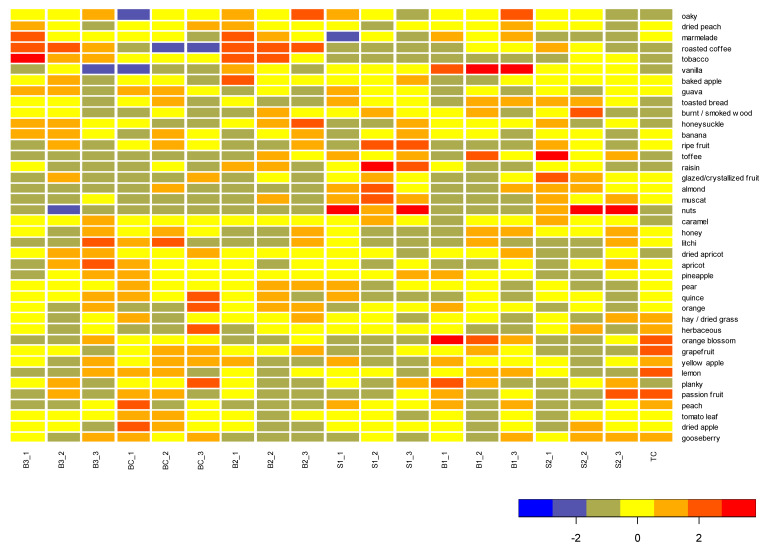
Heat maps constructed based on the standardized residuals that were generated by the correspondence analyses of individual repeats obtained with the trained panel (*n* = 20) after 9 months of oak maturation. The more negative (i.e., the more “blue” on the heatmap) the residuals, the more indication that the correspondence (co-occurrence) between the items are less than what would be expected under the assumption of complete random occurrence. And vice versa, the more positive (i.e., the more red on the heatmap) the residuals, the more indication that the co-occurrence are more that expected under complete random occurrence. TC: Tank control, BC: Barrel control, B: New barrel treatments, S: Stave treatments.

**Figure A4:**
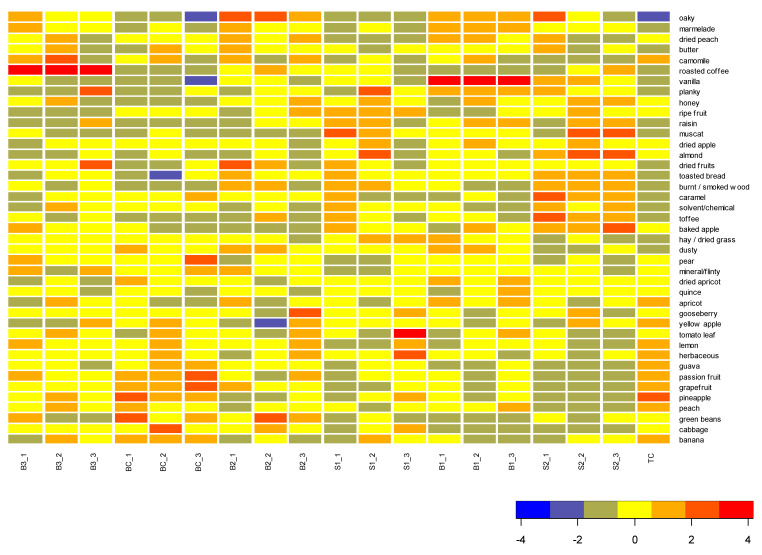
Heat maps constructed based on the standardized residuals that were generated by the correspondence analyses of individual repeats obtained with the trained panel (*n* = 20) after 6 months of bottle ageing. The more negative (i.e., the more “blue” on the heatmap) the residuals, the more indication that the correspondence (co-occurrence) between the items are less than what would be expected under the assumption of complete random occurrence. And vice versa, the more positive (i.e., the more red on the heatmap) the residuals, the more indication that the co-occurrence are more that expected under complete random occurrence. TC: Tank control, BC: Barrel control, B: New barrel treatments, S: Stave treatments.

There were no significant differences (*p* ˃ 0.05) between the different wines in terms of residual sugar, alcohol, total acidity, pH and volatile acidity levels (results not shown). In the following section, CA plots are shown to investigate associations between the treatment repeats and the most prominent wine descriptors at that stage. Separate plots are shown for 4 months and 9 months barrel ageing, as well as the additional 6 months bottle ageing. Heatmaps of the CA standardized residuals are provided in the Appendix A (Figure A2, Figure A3 and Figure A4) as an alternative visual representation of the CA plots.

### 3.1. Chenin Blanc Wine’ s Sensory Profile after 4 Months Oak Maturation

**Figure 1:**
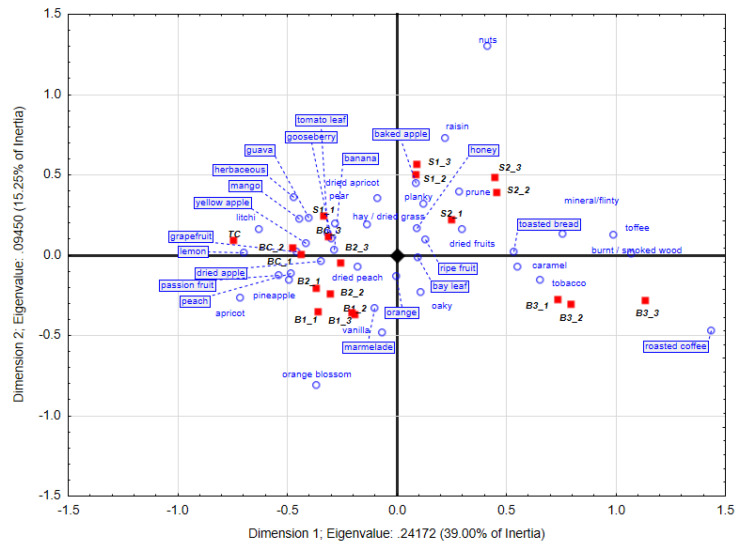
Correspondence analysis obtained with the trained panel (*n* = 20) after 4 months of oak maturation from Pick *K* data. Certain descriptors (boxed) have been moved to improve the readability of the plot. TC: tank control, BC: barrel control, B: new barrel treatments, S: stave treatments.

Wine from the unoaked tank control (TC) was mostly associated with fruit-derived descriptors, which included passion fruit, grapefruit and litchi (Figure 1). The barrel control treatments (BC) were mostly associated with fresh fruit descriptors such as guava, pineapple and passionfruit as well as certain dried fruit descriptors that included dried apple. Some of these are similar to those reported in a study that investigated the effect of inoculated vs. natural fermentation on Chenin blanc wine [7]. In this work the same Chenin blanc wine was also fermented in old barrels and Pick-*K* attributes used to determine the sensory properties of the wine. Descriptors in Chenin blanc such as passion fruit and grapefruit are due to 3-mercaptohexan-1-ol (3MH) and 3-mercaptohexyl acetate (3MHA) that have been proven to occur in wine made from this cultivar [24,25].

The new barrel treatments, B1_1 to B1_3, were associated with vanilla, orange blossom, oak and marmalade. Repeats B2_1 and B2_2 were mostly associated with banana, apricot, dried peach, passion fruit and pineapple aromas. The B3 treatments had roasted coffee, burnt/smoked wood, toffee and caramel characters. It thus seems that the B3 treatments had the most prominent oak aromas extracted at this stage of the barrel ageing process (Figure 1). This might also help to explain the better separation between the B3 vs. B1 and B2 treatments at this stage, with the latter two treatments correlating better with the other treatments with less wood contact (TC and BC).

In terms of repeatability, the S1 stave treatments after 4 months of oak maturation, were not as well defined as that of S2. S1_1 had more gooseberry, tomato leaf and peach aromas, while the other two biological repeats had more raisins, planky and baked apple aromas. Wines treated with S2 associated more with typical wood-derived aromas such as nuts, honey, toffee, burn wood, planky and caramel (Figure 1). A number of factors such as wood composition, seasoning, stave size, toasting procedures and origin of the oak can influence the composition of oak staves and the rate of extraction of oak-derived aromas into wine [26,27,28]. According to a study on Sauvignon blanc and Chardonnay, most volatile compounds playing an important sensory role increased from five to 12 months [11], which was probably the case in our study as well, where large extractions of oak-derived compounds did not take place at this stage.

### 3.2. Chenin Blanc Wine’ s Sensory Profile after 9 Months Oak Maturation

**Figure 2:**
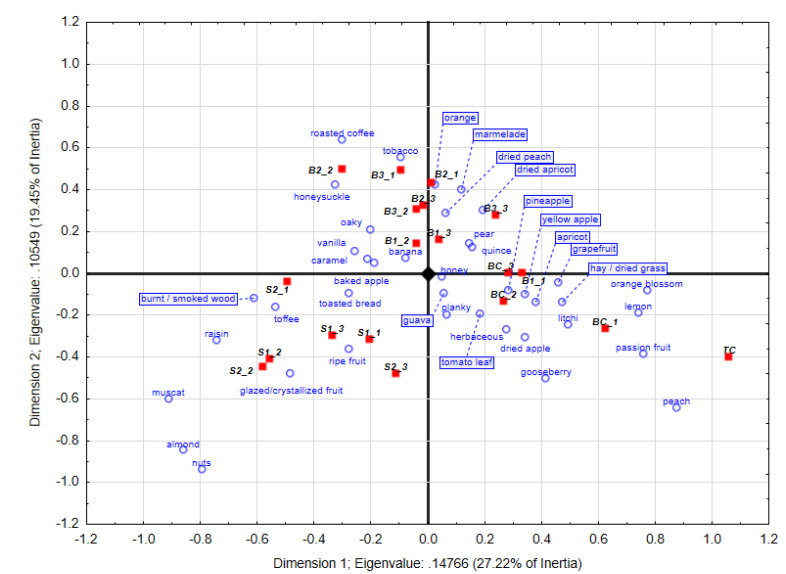
Correspondence analysis obtained with the trained panel (*n* = 20) after 9 months of oak maturation from Pick *K* data. Certain descriptors (boxed) have been moved to improve the readability of the plot. TC: tank control, BC: barrel control, B: new barrel treatments, S: stave treatments.

The main separation of the treatments on F1 of the CA plot occurred at this stage, which accounted for 27.2% of the explained variance (Figure 2), with F2 accounting for 19.4% explained variance.

In Figure 2 a clear discrimination between the TC, BC and the stave treatments on F1 can be seen, with the BC treatment separating better on the F2 axis from the new barrel treatments. The tank control wine was associated more with, lemon, passionfruit, grape fruit, gooseberry and orange blossom descriptors. The barrel control treatments were less repeatable between the three repeats. BC_1 was more associated with peach and dried apple, BC_2 with litchi and BC_3 with planky, orange and quince. Some further variation between the barrel control wines, with cabbage being for instance also more prominent in BC_2 for instance, might be due to slight differences in the chemical composition of these old barrels, although care was taken to select barrels in which the same wines were previously matured. Such slight variations might be due to small difference in extraction by the previous wines, induced by changes in humidity, temperature and amount of yeast lees that might all influence extraction over time [8,28,29] or possibly differences in oxygen transmission through the staves which becomes lower in older barrels [30].

Repeats from the new barrel treatment B1 (B1_1 to B1_3) had especially vanilla and in most cases orange blossom characters, with planky and oak also picked up in certain repeats. The planky aroma can be due to *trans*-2-nonenal, *trans*-2-octanal and 1-decanal, sometimes associated with new French wood [31]. However, the wines matured in B2 and B3 had more toasted-derived aroma descriptors, such as roasted coffee, which could be due to Furfurylthiol [32] and tobacco at this stage. Honey suckle (B2_2 and B2_3) and marmalade (B3_1 and B3_2) were also associated with these repeats at this evaluation stage. The clear separation of the BC wines from those matured in new barrels is due to the leaching effect of successive wines being matured in the same barrel, leading to much lower levels of oak-derived aroma compounds ending up in the wine after barrels have been used for a number of times [33].

Both stave treatments were strongly associated with certain wood-derived aromas at this stage, such as nuts, almond and toffee (especially biological repeats 1 and 3 in both treatments). Caramel was also prominent in the S2 treatment. These descriptors are derived from toasting of the wood, and can be due to furan aldehydes formed during toasting. Muscat, a grape-derived descriptor originating from terpenes were picked up in both these treatments, with ripe fruits also being prominent in most stave treatment repeats. It also seems that the S1 repeats were more closely associated than the S2 treatments at this stage.

### 3.3. Chenin Blanc Wine’ s Sensory Profile after 6 Months of Bottle Ageing

**Figure 3:**
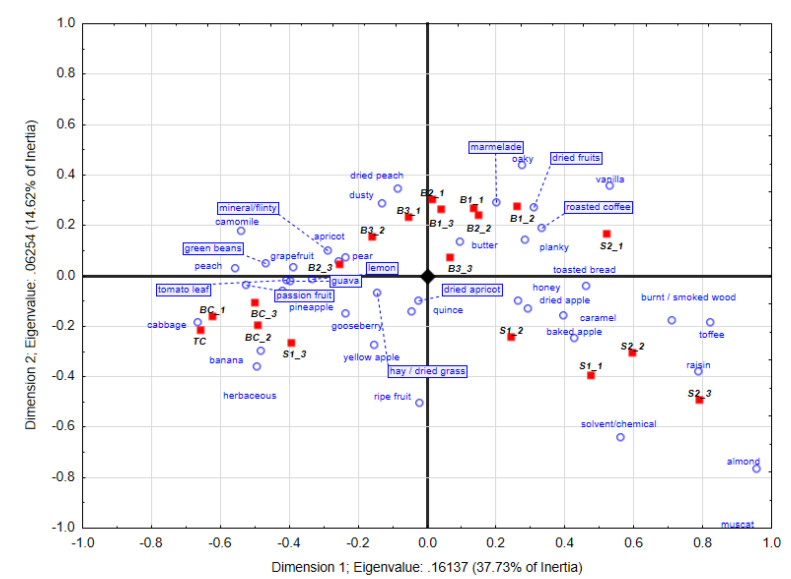
Correspondence analysis obtained with the trained panel (*n* = 20) after 9 months of oak maturation and 6 months bottle ageing from Pick *K* data. Certain descriptors (boxed) have been moved to improve the readability of the plot. TC: tank control, BC: barrel control, B: new barrel treatments, S: stave treatments.

A cumulative variance of 52.3% can be seen in the CA plot, with F1 and F2 explaining 37.7% and 14.6%, respectively, of the variance (Figure 3). Separation of the treatments was observed on F1, with the wines receiving the least amount of wood treatments (TC and BC), new barrel treatments and stave treatments separating mainly from each other. On F2 most of the stave repeats separated from the new barrel treatments. The barrel control wines (BC) and tank control (TC) wines were associated mostly with fresh fruit aromas including pineapple, grapefruit, passionfruit and banana. The TC samples also further associated with guava, lemon and apricot, while green beans (repeat 1 and 3) and cabbage in repeat 2 of the BC treatments were also more prominent. It thus seems that the old oak barrels used in the BC treatments did not impart higher levels of oak-derived aroma notes to the wines, which was still reflected after 6 months of bottle ageing.

The wines matured in the B1 new oak barrels treatments were related to oaky, vanilla, planky, and marmalade in all three repeats, while apricot and dried apricot were more prominent in repeats 1 and 3 and 1 and 2, respectively. The B2 treatments had oaky, burnt smoke, dried fruits (repeats 1 and 2) and gooseberry in repeat 3. Roasted coffee was especially still prominent in treatment B3 at this stage as well. Regarding the stave treatments, S2_1 to S2_3 were associated with toffee, caramel, baked apple and honey aromas. Regarding the S1 treatments, these were associated with ripe fruit and muscat, raisins and burned wood in repeats 1 and 2. S1_3 was separated the furthest on F2 and had herbaceous-associated characters such as tomato leaf, and lemon aroma descriptors. A trend was seen that that the number of attributes used by the panel to describe the aroma profiles of the barrel control wines became more over time (results not shown), which might indicate that the wines became more complex with ageing.

In general, no oak-related descriptors were used to describe the tank control wine at the three intervals investigated. The conclusion can thus be made that the panel considered the tank control as being unoaked. As with the unoaked tank control (TC), the influence of oak in the barrel control wines (BC) were considered minimal. This is due to the oak-derived aroma compounds being diminished through leaching during previous fills [30]. The effect of the staves was clearly detectable in the treatments where these were added to the old barrels. However, the old barrel control wines differed considerably from the stave treatment wines, showing the effect of the latter treatments.

After 4 months of oak ageing, the panel considered both stave treatments as being associated with planky aromas, with the S2 treatment associating with additional oak-derived descriptors. However, after 9 months of ageing, the profiles of the two stave treatments were similar, being associated with caramel, toffee, nuts, and muscat characters. This might indicate that the possible initial higher extraction suggested by the S2 treatment after 4 months ageing was negated to an extent. Some of these aromas found after 9 months maturation persisted in the 6-month bottle aged wines as well.

Looking at the new oak barrel treatments, grouping of some of the biological repeats were seen after 4 months oak ageing, with one of the treatments having a strong roasted coffee, smoked wood character in all three biological repeats, with another treatment having a prominent vanilla aroma. However, some of these large differences were negated to an extent after 9 months oak maturation and 6 months bottle maturation, indicating that the cooper effect or differences in the rate of extraction of new barrels decreased to an extent after a certain period, when compared to the other treatments. The poorer discriminability between different coopers can also be considered a possible drawback of the CATA approach, since it does not indicate specific quantitative differences. However, CATA has also been successfully used to investigate the effect of oak chips on the sensorial composition of Shiraz wine [19]. After the 6 months of bottle ageing, all the wines matured in new barrels had prominent wood-derived aroma profiles, with a loss in fruitiness compared to those matured in old barrels (BC). Perez-Prieto et al. [29] reported that wine matured in old barrels retained more fruitiness, due to it having a lower porosity, and thus resulted in less evaporation. The time wine spend in a barrel seems to be one of the most important factors affecting how much oak-derived aroma compounds leach into the wine, which might mask other fruity aromas. Du Toit et al. [16] and Prida and Chatonnet [34] have also reported that fruitiness in wine can be masked by oak maturation over time, with furanic aldehydes thought to be responsible for this [26]. Formation and later acid hydrolysis of fruit-associated compounds such as esters during barrel ageing has also been found [26]. Varietal thiols, responsible for the guava and passion fruit aromas in Chenin blanc [25], have also been reported to be sensitive towards acid hydrolyses over time [3]. These factors might all have played in a role in the changes observed in the sensory profiles of the Chenin blanc during the bottle and barrel ageing periods.

Although no chemical analyses were performed to compare and quantify differences in the levels of the oak-derived compounds in the different treatments, clear differences in the descriptors used in the new barrel and the stave treatments were found. In general, the treatments separated according to the type of wood treatments, with the TC and BC associating more, the stave treatments with each other and the new barrel treatments with each other, which were reflected during both barrel and bottle maturation. Studies have showed that different extraction kinetics can exist between oak barrels and oak alternatives such as staves and chips [16,27]. It is well known that staves destined for alternative oak products are often not produced using the same quality of wood and method as those used for barrel production. The type and composition of oak product can thus have a significant influence on the extraction of oak-derived aromas into the wine [16]. This could be due to significant differences in the chemical composition, such as *cis*-oak lactones, of oak-derived alternative products such as staves compared to those of barrels [16]. This could further explain the large difference observed in our study when the new barrel and stave-treated wines were compared. However, little differences in oak lactone levels [16] in wine as well as sensorial differences in brandy [35] were reported when these alcoholic products were matured in staves and barrels that were produced from the same batch of wood, possibly further corroborating this hypothesis.

This work shows the potential of alternative oak products for wine producers to produce Chenin blanc wines of a different aromatic composition than those matured in oak barrels, which can still be reflected after a period of bottle maturation. However, this outcome will depend on the composition of the alternative oak product, as well as the time spent in contact with it. If an alternative oak product such as staves is used instead of new barrels, but a similar sensorial outcome is required, wine producers should re-assess the origin and composition of these alternative products.

Finally, differences between repeats in our work has shown the variability between different oak products, even among repeats of the same treatment. This was also reported by other authors [8,11] for the chemical analyses of white wines matured in the same barrels, although in the latter work the repeats of a treatment were pooled for sensorial analyses. This should be kept in mind in future work focusing on the chemical and sensorial composition of wines matured in contact with different wood products over time.

## 4. Conclusions

This study investigated the effect of different oak treatments on the aroma profile of a commercial scale Chenin blanc wine at three different intervals of maturation. Differences were observed between the main treatments at these maturation intervals (after 4 and 9 months of wood maturation and an additional 6 months of bottle maturation). Clear differences were observed between the unoaked and the oaked wines, and also among the different oak treatments. This included a clear discrimination between the wines matured in the old barrels with staves with those in new barrels. The rate of release of oak-derived compounds between different wood products seems to differ, which can affect a wine’s sensorial composition, with longer maturation leading to a more oaky aroma profile. A clear separation between barrels and staves in general can also occur. Longer oak contact can also lead to a loss of fruitiness, which can further affect the wine’s organoleptic characteristics. Wine producers could thus use barrels or staves as well as time spent in contact with the wood, to obtain a different outcome in terms of the sensory composition of Chenin blanc wine.

It is recommended that chemical analysis and other sensory descriptive analysis or rank-all-that-apply (RATA) methods are included in future, to better qualify and quantify differences between the different oak treatments over time. The appropriate biological repeats for both these types of analyses are, however, recommended.

## Figures and Tables

**Table 1:** Wood treatments (*Quercus petraea*) used for the maturation of Chenin blanc wines.

Treatment and Volume/Dimension	Abbreviation	Repeats
Tank control (2000 L)	TC	Only one tank, no repeats
Old barrels (300 L)	BC	BC_1, 2 and 3
New barrel cooper 1 (300 L)	B1	B1_1, 2 and 3
New barrel cooper 2 (300 L)	B2	B2_1, 2 and 3
New barrel cooper 3 (300 L)	B3	B3_1, 2 and 3
Old barrels with staves cooper 4 (300 L)	S1	S1_1, 2 and 3
Old barrels with staves cooper 5 (300 L)	S2	S2_1, 2 and 3

## Appendix A

**Table A1:** Aroma attributes and the composition of the reference standards presented during panel training.

Aroma Family	Sensory Attribute	Reference Standard Composition	Amount
Citrus	Lemon	Fresh lemon *	¼ slice
	Orange	Fresh orange *	¼ slice
	Grapefruit	Fresh grapefruit *	¼ slice
White/Yellow fruit	Peach	Peach juice (Liquifruit)	20 mL
	Apricot	Apricot juice (Liquifruit)	20 mL
	Melon	Fresh melon *	1 cm^3^ piece
	Pear	Canned pear (Koo)	2 × 3 cm piece
	Quince	Fresh quince *	1 cm^3^ piece
	Yellow apple	Fresh apple (golden delicious) *	1 cm wedge
Tropical	Banana	Isoamyl acetate (Merck) + 30mL distilled water	1 µL
	Litchi	Canned litchi syrup (Pot’O Gold)	5 mL
	Passion fruit	Fresh passion fruit pulp and 4 pips *	5 mL pulp
	Pineapple	Fresh pineapple *	4 cm^3^ piece
	Guava	Fresh ripe guava *	2 × 3 cm^3^ pieces
	Mango	Dried mango roll (Montague)	2 cm^2^ square
	Gooseberry	Gooseberries^-^(Hillcrest)	2
	Papaya	Fresh papaya *	1 cm wedge
	Coconut	Desiccated coconut (Imbo)	5 mL
Vegetative	Fresh cut grass	Freshly cut grass	A handful
	Green beans	Fresh green beans (chopped) *	2
	Green pepper	Green pepper (chopped) *	2 × 3 cm piece
	Mint	Fresh mint (crushed) *	1 sprig
	Eucalyptus	Eucalyptus leaves (crushed)	5
	Celery	Celery stick (chopped) *	1 × 10 cm
Dried	Dry grass/Hay	Dry grass (local petshop)	A handful
	Tobacco	Dried tobacco (Peter Stuyvesant)	2 cigarettes
Sweet Associated	Honey	Acacia honey (Luna de Miel, France)	5 mL
	Marmalade	Ceville orange marmalade (Koo)	5 mL
	Dried fruit	Dried apple, apricot, peach, prune, pear-chopped (Safari)	1 piece each
	Raisin	Raisins-chopped (Safari)	5
Nutty	Nutty	Walnut flavour (MANE) + 10 mL distilled water	2 drops
Floral	Orange blossom	Orange blossom flavour on cotton wool (Vanhiné)	2 drops
	Linden Tree Flower	Liden tree tea (Twinings)	½ teabag
Toasted/Oaky	Oak	Medium toasted French oak (NT Bois, RX South Africa)	3 g
	Planky	Pine wood shavings (Timber city)	3 g
	Toasted bread	Toasted white bread (Sasko)	1 cm^3^
	Caramel	Caramel sauce (Ilovo) + 5 mL distilled water	5 mL
	Toffee	Ttoffee chopped (Toff-O-Lux) + 5 mL hot water	1
	Vanilla	Vanilla essence (Robertson) in 10 mL distilled water	2.5 mL
	Roasted Coffee	Roasted coffee beans (LavAzza)	4
Spicy	Black pepper	Black pepper—grinded (Robertson)	5 mL
	White pepper	White pepper (Robertson)	5 mL
	Bay leaves	Dry bay leave (Robertson)	1
	Cinnamon	Cinnamon powder (Robertson)	2.5 mL
	Thyme	Dry thyme (Robertson)	2.5 mL
	Cloves	Cloves (Robertson)	2.5 mL
	Juniper	Juniper berries (Robertson)	4
	Aniseed	Whole aniseed (Robertson)	2.5 mL
	Nutmeg	Ground nutmeg (Robertson)	2.5 mL
	Ginger	Ginger powder (Robertson)	2.5 mL
	Liquorice	Liquorice (Mister sweet)	1 cm piece
	Curry	2.5 mL curry powder (Rajah)	2.5 mL
Forest floor	Mouldy/mushroom	Fresh mushroom in 10 mL distilled water	½ mushroom
	Earthy	Wet soil	25 mL
Other	Butter/lactic	Butter (Clover)	2 cm^3^
	Sulphur	SO_2_ solution (Protea) in 15 mL distilled water	15%
	Yeast	Rehydrated yeast (VIN 13, Anchor)	20 mL

* Fresh reference standards were purchased from the local grocery store.
